# QuantiFERON^®^ Monitor Test as a Potential Tool for Stratifying Patients by Infection Risk and Tailoring Follow-Up Care in Lung Transplant Recipients: A Single-Center Retrospective Experience

**DOI:** 10.3390/microorganisms13020316

**Published:** 2025-02-01

**Authors:** Paolo Solidoro, Antonio Curtoni, Filippo Patrucco, Eleonora Russo, Francesca Sidoti, Giorgia Piccinini, Alessandro Bondi, Paolo Valesella, Mattia Genco, Massimo Boffini, Rocco Francesco Rinaldo, Cristina Costa

**Affiliations:** 1 Cardiovascular and Thoracic Department, Division of Respiratory Medicine, AOU Città della Salute e della Scienza di Torino, 10126 Torino, Italy; psolidoro@cittadellasalute.to.it (P.S.); russo.el@ospedale.cuneo.it (E.R.); roccofrancesco.rinaldo@unito.it (R.F.R.); 2 Medical Sciences Department, University of Turin, 10126 Torino, Italy; 3 Microbiology and Virology Unit, Department of Laboratory Medicine, University Hospital Città della Salute e della Scienza di Torino, 10126 Turin, Italy; antonio.curtoni@unito.it (A.C.); francesca.sidoti@unito.it (F.S.); alessandro.bondi@unito.it (A.B.); paolo.valesella@unito.it (P.V.); cristina.costa@unito.it (C.C.); 4 Department of Public Health and Paediatrics, University of Turin, 10126 Turin, Italy; giorgia.piccinini@unito.it (G.P.); mattia.genco@unito.it (M.G.); 5 Respiratory Diseases Unit, Medical Department, AOU Maggiore della Carità di Novara, 28100 Novara, Italy; 6 PhD National Programme in One Health Approaches to Infectious Diseases and Life Science Research, Department of Public Health, Experimental and Forensic Medicine, University of Pavia, 27100 Pavia, Italy; 7 Cardiac Surgery Division, Surgical Sciences Department, AOU Città della Salute e della Scienza di Torino, University of Turin, 10126 Torino, Italy; massimo.boffini@unito.it

**Keywords:** QuantiFERON Monitor Test, lung transplantation, immunosuppression

## Abstract

Background: Lung transplantation is a life-saving option for patients with end-stage respiratory diseases, but risk of infections remains critical for ensuring long-term organ function. This study aimed to assess immune recovery in lung transplant recipients by measuring IFN-γ levels using the QuantiFERON Monitor Test (QFM). Results were correlated with episodes of infection and organ rejection to explore the assay’s predictive potential. Methods: A retrospective study was conducted on 15 lung transplant recipients at the Lung Transplant Centre of Turin (Città della Salute e della Scienza di Torino, Italy) between December 2019 and January 2023. Patients were divided into a High Infection (HI) group (with >3 infections) and Low Infection (LI) group (with ≤3 infections). QFM assays were performed after 18 months post-transplant. Results: HI patients had lower QFM levels compared to LI (68.84 ± 21.98 vs. 380.54 ± 104.64 UI/mL, *p* = 0.033). A QFM value <89.5 UI/mL was associated with increased infection risk (*p* < 0.05). Patients with lower QFM levels also exhibited higher rates of MRSA bacteremia during hospitalization (50% HI vs. 0% LI, *p* = 0.04). No differences were observed in acute or chronic rejection rates, but LI patients showed more frequent alveolar neutrophilia at the fourth month post-transplant (0% HI vs. 55.5% LI, *p* = 0.04). Conclusion: lower QFM values were associated with higher infection risk, highlighting the assay’s potential for immune monitoring. In this study, a QFM value of 89.5 UI/mL showed good predictive accuracy for infections beyond 18 months. Further studies are needed to refine QFM’s role in post-transplant care.

## 1. Introduction

Single or bilateral lung transplantation is a therapeutic option in patients suffering from end-stage non-oncological respiratory diseases with irreversible damage, because conventional therapies are no longer sufficient to ensure a good life quality and when there is low life expectancy [1]. Respiratory diseases that currently require a lung transplant can be divided into three groups: diseases involving the airways, lung parenchyma and those affecting the vascular bed [2].

Despite all the improvements introduced to the transplant procedure, the correct balance between immunosuppression and increased infectious risk is still a delicate objective to be pursued for the proper functioning of the organ in the short and long term.

The immunosuppressive therapy is based on two strategies: induction and maintenance [3]. The first is based on the use of T-cell inhibitors or non-T-cell inhibitor drugs [4,5]. The second aims to reduce long-term pharmacological toxicity related to the use of immunosuppressants with therapeutic strategies that currently include the combination of three drugs: a glucocorticoid, a calcineurin inhibitor (cyclosporine or tacrolimus) and an inhibitor of purine synthesis (azathioprine, mycophenolate mofetil or mycophenolate sodium) [6,7].

Regarding infections, the anti-infective prophylaxis is started already in the pre-transplantation phase and then it continues in the peri- and post-transplantation phases. Monitoring the specific or nonspecific immune response is fundamental in the post-transplant period, as there is a balance between immunodepression to avoid rejection and immunocompetence to combat infections. The specific immune response to CMV [8,9,10], Mycobacterium tuberculosis [11,12,13], Herpesvirus [14,15] and SARS-CoV-2 [16,17] is now tested by measuring interferon-γ produced by T-cells after stimulation with a specific antigen. The interferon-γ data can predict the immune response, but it is necessary to standardize the results in all laboratories.

Experimental strategies to evaluate the nonspecific immune response have different objectives, such as measuring immunoglobulin levels after transplantation, which correlate with infections by capsulated bacteria [18,19], or complement factor dosage [20], especially C3, which also correlates with an increase in bacterial infections. Furthermore, lower levels of CD4+ and C8+ are associated with CMV-related post-transplant viral [21] and P. jiroveci infections [22]. The main limitation of these strategies is the lack of a cut-off to divide patients into two groups, high and low risk of infection.

The only FDA-approved assay is ImmuKnow (Cylex Inc., Columbia, MD, USA) [23], which can measure CD4+ activity after nonspecific stimulation in relation to plasma ATP levels. The assay predicts acute rejection with a high specificity but low sensitivity [24], and its role in predicting infections is too variable and dependent on the type of pathogen.

Quantiferon Monitor Test (QFM) (QIAGEN, Hilden, Germany) provides assessment of an individual cell-mediated immune response through dual innate and adaptive immune system stimulation. It measures interferon gamma (IFN-γ) production in plasma after incubation of heparinized whole blood with innate (R848, a TLR7 agonist) and adaptive (CD3 antibody) stimulants. The result of the assay allows the classification of patients into three groups that correlate with the intensity of immune activity: low <15 UI/mL, intermediate 15–1000 UI/mL and high >1000 UI/mL [25].

The potential benefits of QFM assay have been previously demonstrated in liver transplant recipients with an IFN-γ increase over time post-transplant and a correlation between assay levels and infections in post-solid-organ transplants [26]. QFM levels are lower after transplantation and at the beginning of immunosuppressive therapy but increase steadily thereafter. QFM is not correlated with sex and age, and there is no statistical association with tacrolimus and cyclosporine immunotherapy [27].

As reported in the literature, a correlation between low QFM values and significant CMV viremia (>1000 UI/mL) has been found in bone marrow transplantation. In contrast, no correlation between QFM and graft-versus-host disease has been found [28].

A statistical association between low QFM values and prednisolone and tacrolimus use has been reported in the lung transplant setting [29]. No associations were found with the risk of infection, but patients with <10 UI/mL QFM values at three months and <60 UI/mL at six months after transplantation have a higher likelihood of infectious disease [29].

The aim of this study is to evaluate IFN-γ levels by using the QFM assay in lung recipients over the course of their transplant to profile immune recovery and correlate results with episodes of infection and organ rejection. These results could predict the risk of CMV infection controlling early viral infection or reactivation in recipients of lung transplant. We hypothesized that IFN-γ levels would be lower in patients who are more immunosuppressed and, therefore, be more susceptible to infections.

## 2. Materials and Methods

### 2.1. Population and Characteristics

Nineteen patients who underwent lung transplant at the Lung Transplant Centre of Turin (Città della Salute e della Scienza di Torino, Italy) between December 2019 and January 2023 were enrolled (Ethics committee protocol n. 0004577-CS/416).

Inclusion and exclusion criteria for study participants were as follows:

- Inclusion criteria: patients undergoing single or bilateral lung transplantation with a follow-up of at least 18 months at enrollment;
- Exclusion criteria: deceased patient, lung re-transplantation, functional or geographical limitation that hinders reaching the transplant center for carrying out the test and patients treated with a single immunosuppressant.

One patient was excluded for refusing study participation and two patients died during the follow-up period. Finally, one patient was excluded due to Posterior Reversible Encephalopathy Syndrome (PRES) occurring after reducing immunosuppressive therapy during follow-up.

Therefore, 15 patients were included in this study and their clinical course was followed longitudinally for 18 months post lung transplant.

Post-transplantation infection prevalence in our population, expressed as the number of infections per patient, was evaluated in the first 18 months of follow-up, considering both lung and extra-lung infections.

In the absence of standardized literature indication, we decided to split the population into two groups in relation to the number of infections during the observation period.

According to our case history, based on more than 20 lung transplantations per year, the groups were defined as follows:

(1) Group 1 (High Infection, HI) patients with a high number of infections (greater than 3);
(2) Group 2 (Low Infection, LI) patients with a low number of infections (less than or equal to 3).

Clinical information, including clinical course, graft functional state via spirometry, CMV infections, compatible histology with acute rejection by performing transbronchial biopsies (TBB), microbiological research or cellular alterations on BAL and blood immunosuppressant dosage (ciclosporin, tacrolimus and everolimus), were collected for each subject at 1, 4, 8, 12 and 18 months post lung transplant. CMV infections were identified by PCR real-time detection CMV DNA (CMV ELITe MGB^®^ Kit, ELITechGroup, Turin, Italy) on whole blood and bronchoalveolar lavage (BAL) samples. At the end of each follow-up visit, immunosuppressive therapy was reassessed based on the blood dosage or indirectly with laboratory tests (blood count and renal function).

### 2.2. QuantiFERON Monitor^®^ ELISA

Venous blood samples of patients were collected for QFM assay after the 18th month of follow-up. QFM is a new diagnostic test able to detect immune activity by measurement of IFN-γ in plasma samples by enzyme-linked immunosorbent assay (ELISA). IFN-γ is a cytokine produced by T cells after incubation of patient whole blood with response-specific stimulants.

QFM assay was performed according to manufacturer’s instructions. Briefly, 1 mL of whole venous blood was collected into the specific heparinized tube provided (QFM Blood Collection Tube, QIAGEN), after pelleted lyophilized inductors anti-CD3 and R848 (QFM LyoSpheres, QIAGEN) [27] were added to the sample, as soon as possible and not later than 8 h after collection. The sample was incubated at 37 °C for 16–24 h; then, the tube was centrifuged for 15 min at 2000–3000× *g*. The plasma was stored at −20 °C. The samples were thawed and the ELISA assay was performed to measure IFN-γ in the same session.

As the results of the QFM assay are both qualitative and quantitative, it is possible to divide the immune function into three groups: low <15 UI/mL, moderate between 15 and 1000 UI/mL and high >1000 UI/mL [25]. To analyze the results of the samples, it is necessary to construct a standard curve and compare the IFN-γ concentration of the samples log(e) (y-axis) and the IFN-γ concentration of the standard log(e) (x-axis) for best fit by regression analysis.

### 2.3. Statistical Analysis

Statistical analysis was performed using IBM SPSS software (version 29.0.2.0). The statistical description was presented as mean and standard error for continuous variables, whereas frequency and percentage were used for categorical variables. The association between categorical variables was assessed by Chi-squared test with Yates correction (for numbers >50 samples) or Fisher test (for numbers <50 samples). The *t*-student test was used to compare continuous variables. For all tests, the significance level was set at 0.05. Finally, to identify a predictive cut-off for the risk of infection after 18 months, a Receiver Operating Characteristic (ROC) was performed.

## 3. Results

### 3.1. Population

This study divided patients into two groups based on the number of infections that occurred during the first 18 months post-transplant:

- HI: patients with more than three infections (six patients);
- LI: patients with three or fewer infections (nine patients).

Groups were compared by age, sex, disease that had led to transplantation and immunosuppressive therapy set in the post-transplantation period. There were no significant differences in age, underlying disease leading to transplantation or immunosuppressive regimens between groups except for sex; a higher prevalence of females was noted in HI (five women, 83%) compared to LI (two women, 22.2%, *p* = 0.04).

The mean number of infections also showed differences between groups (*p* < 0.001), with an average of approximately eight infections in HI and two infections in LI.

Furthermore, the groups were analyzed based on the type of immunosuppressive agents used, such as calcineurin inhibitors (Immunosuppressor 1: cyclosporine or tacrolimus) and purine inhibitors (Immunosuppressor 2: Mycophenolate Mofetil-MMF or Mycophenolate Sodium-MS). No statistically significant differences were observed in these parameters (*p* > 0.05) (Table 1).

### 3.2. Comparison Between Groups Excluding CMV Infection

**Figure 1:**
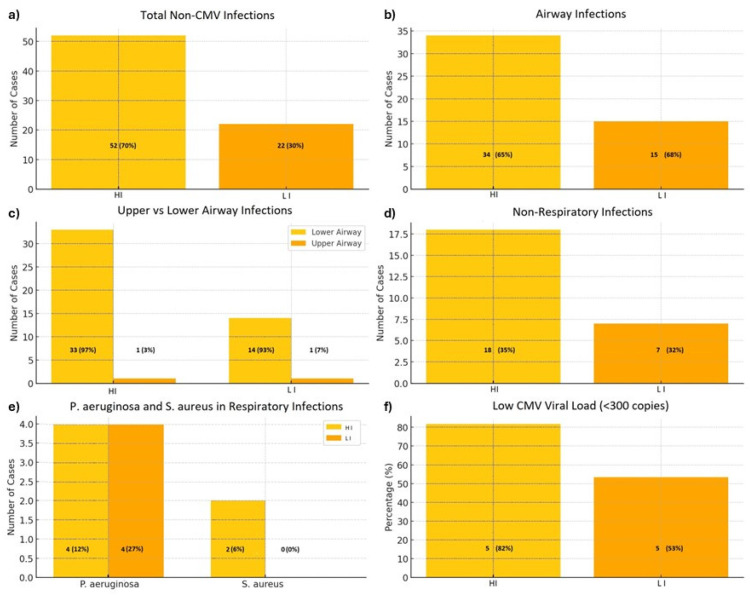
Comparison between HI and LI groups in the onset of viral and bacterial infections: (**a**) cases of non-CMV infections; (**b**) cases of airways infections; (**c**) cases of airways infections divided in lower and upper tract; (**d**) cases of non-respiratory infections; (**e**) cases of the principal bacterial isolated species; (**f**) percentages of patients with CMV viral load <300 copies/mL on BAL.

The QFM assay was performed after 18 months. The overall incidence of respiratory and non-respiratory infections was assessed, excluding those caused by CMV. HI experienced 52 non-CMV-related infections (70%), while LI had 22 such infections (30%) (Figure 1a). Cases of non-CMV viral infections were six (12%) in HI vs. three (14%) in LI.

A statistically significant difference emerged between the two groups (*p* = 0033), with an average value of 68.84 ± 21.8 UI/mL in HI compared to 380.54 ± 104.64 UI/mL in LI.

Among the infections in HI, 34/52 (65%) were respiratory, compared to 15/22 (68%) in LI (*p* = 0.816) (Figure 1b). Among these, infections of the upper airways were rare, with just one case in each group. Most respiratory infections involved the lower airways: 33/34 cases (97%) in HI and 14/15 cases (93%) in LI (*p* = 0.543) (Figure 1c).

Non-respiratory infections occurred in 18/52 cases (35%) in HI and 7/22 cases (32%) in LI, with no significant statistical differences (Figure 1d).

The type of infection, healthcare settings where infection occurred and microorganism isolated from non-CMV-related infections were examined.

In terms of infection types, pneumonia and isolates obtained via BAL were equally common across groups, with no significant differences. Similarly, acute bronchitis and anastomotic pseudomembranes showed comparable rates between groups. The distribution of pathogens responsible for respiratory infections varied but lacked statistically significant differences. Notably, *Pseudomonas aeruginosa* was the most frequently identified pathogen, with group 1 showing a slightly higher prevalence (Figure 1e). *Staphylococcus aureus* appeared exclusively in HI (Figure 1e). Infections caused by other pathogens such as *Aspergillus*, *Klebsiella pneumoniae*, *Escherichia coli*, *Moraxella catarrhalis*, *Streptococcus pneumonia*, *Corynebcterium*, *Achromobacter*, *Enterobacter*, *Serratia*, and HSV-1 were observed in both groups at varying rates without statistically significant differences.

Non-respiratory infections also showed similar patterns, with blood cultures, urinary tract infections and intertrigo as common sites. However, a notable finding was the significantly higher incidence of *Staphylococcus epidermidis* (MRSE) bacteremia (3/5, 50%) in HI compared to LI (0/1) (*p* = 0.044).

CMV-related infections, including pneumonia, were absent in both groups and viral load analysis in BAL samples revealed a higher prevalence of low loads (<300 copies/mL) in HI (82%) compared to LI (53%) with a statistically significant difference (*p* = 0.046) (Figure 1f).

Chronic rejection did not occur in either group during the 18-month follow-up. Acute rejection requiring steroid bolus treatment (grade >A2) was observed in 33% of LI patients but not in HI (*p* = 0.514). Both groups followed similar immunosuppressive regimens, with no significant differences in corticosteroid use.

### 3.3. Comparison Between Groups at the Follow-Up

The two groups were compared at the periodic follow-up conducted at 1, 4, 8 and 18 months. Key parameters included CMV incidence in whole blood and BAL, neutrophilia (>3%) in BAL, histological findings of acute rejection (grade A2 or higher) and the presence of galactomannan antigen (>0.5 S/CO). There were not statistically significant differences between the groups across most follow-up intervals, except at the fourth month.

At the fourth month of follow-up, a detailed analysis of BAL and whole blood samples revealed key insights into differences between the two groups.

**Figure 2:**
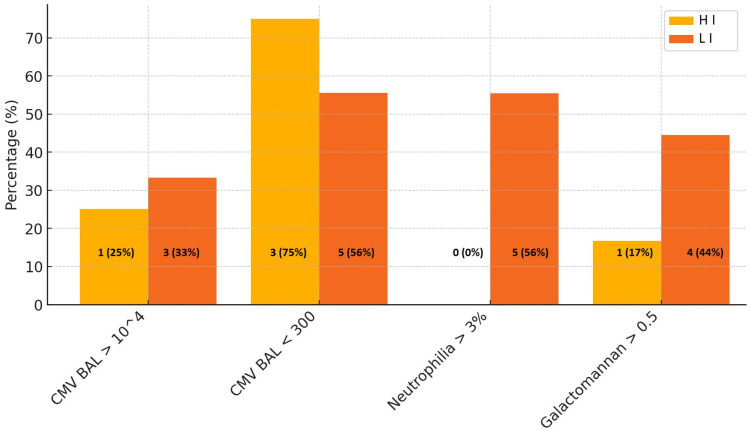
Key BAL results at the 4th month: comparison between HI and LI groups in the percentages of cases with CMV BAL viral load (copies/mL), neutrophilia (%) and galactomannan dosage (S/CO).

Furthermore, LI exhibited a notably higher prevalence of neutrophilia in BAL samples, with five patients affected (56%) compared to none in HI (*p* = 0.04) (Figure 2). This suggests a potential difference in inflammatory responses between the two groups.

For CMV detection in BAL samples, the rates of high viral loads (>10⁴ copies/mL) and low viral loads (<300 copies/mL) were comparable between the groups, with no statistically significant differences observed (*p* = 1.00) (Figure 2). Similarly, the average viral loads for both CMV and Epstein–Barr virus were higher in LI, but the differences did not reach statistical significance.

In the whole blood sample analysis for CMV, no significant disparities were observed between the groups in terms of high viral loads (>10⁵ copies/mL) or low viral loads (<300 copies/mL). This consistency highlights similar systemic viral dynamics between the two groups at this time point.

Regarding galactomannan antigen levels, both the mean levels and the rates of positivity (>0.5 S/CO) were similar in the two groups, with no significant differences identified (Figure 2).

**Figure 3:**
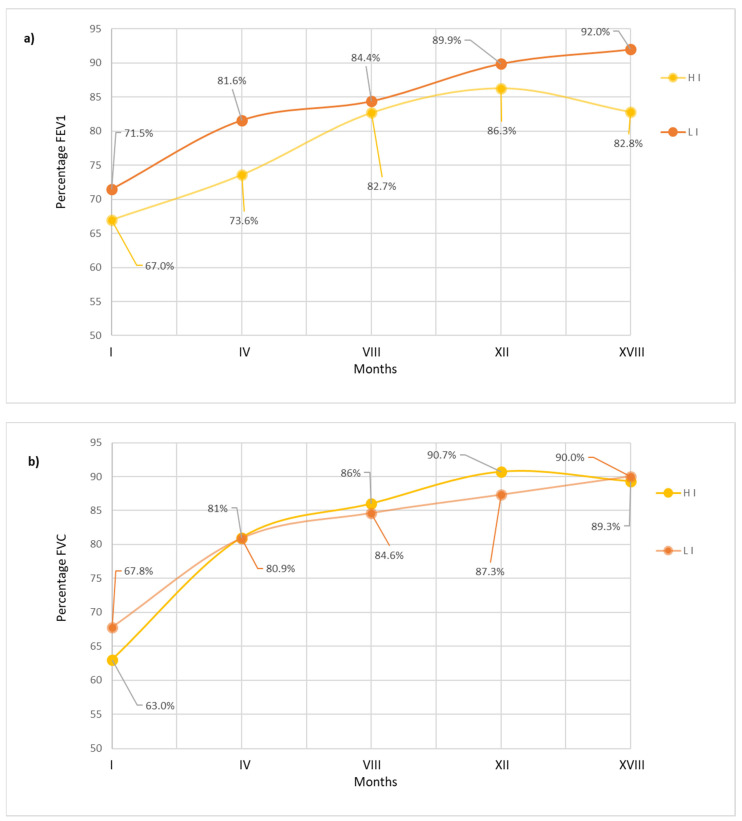
(**a**,**b**) Lung function trends over time: comparison between HI and LI groups in terms of percentages of FEV_1_ and FVC.

Lung function was monitored at each follow-up using forced expiratory volume in the first second (FEV₁) and forced vital capacity (FVC) values. Both groups showed consistent improvements in lung function over time and no statistically significant differences were detected at any time point (Figure 3a,b).

Changes in immunosuppressive therapy and their underlying reasons, such as kidney failure, neoplasia or recurrent infections, were documented. One in six patients in HI (17%) reduced therapy at the 8th month due to kidney failure. In LI, 2/9 patients (22%) modified therapy to include everolimus due to neoplasia. There were no significant differences between the groups regarding therapy adjustments or reasons for these changes.

### 3.4. Correlation Analysis of QuantiFERON Monitor Value

Considering the study population (15 patients), we evaluated the correlation between the QFM value and other variables (age, acute rejection A2-TBB, neutrophilia identified in BAL and whole blood concentration of prednisone), but there were not statistically significant differences.

**Figure 4:**
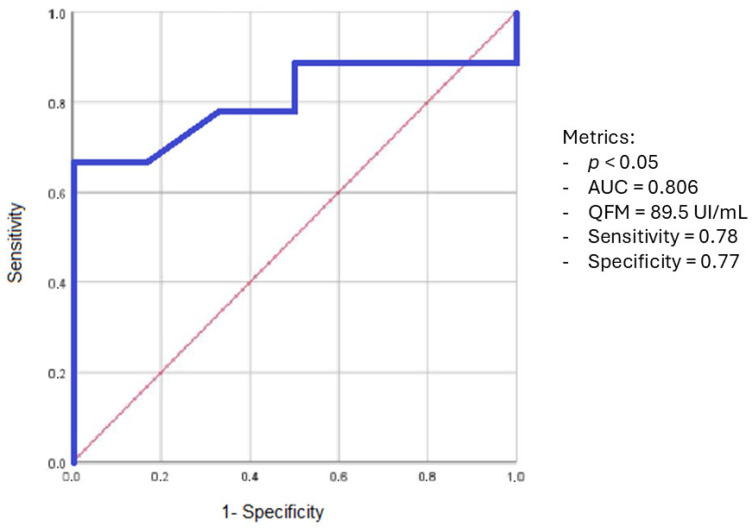
ROC curve: QFM sensitivity and specificity for the risk of infections after 18 months post-transplantation.

From the ROC analysis, as regards the risk of infections after 18 months post-transplantation, we obtained a sensitivity of 78% and a specificity of 77% for QFM cut-off values <89.5 UI/mL (Figure 4).

## 4. Discussion

This study assessed the utility of the QFM assay in evaluating the non-antigen-specific cell-mediated immune response in immunocompromised patients after lung transplantation. Specifically, the aim of this study was to determine whether QFM values correlate with the incidence of infectious diseases or graft rejection. By focusing on QFM values measured 18 months post-transplantation, the test’s ability was examined to predict complications while remaining independent of the effects of immunosuppressive therapy.

Our results confirm those previously reported by Gardiner et al. in lung-transplanted patients but we had a longer follow-up [29]. Our results indicated that patients with lower QFM values experienced a higher incidence of infections within the first 18 months post-transplantation, despite receiving similar immunosuppressive regimens. This suggests that QFM identifies a baseline susceptibility to infection likely driven by individual genetic or other intrinsic factors. Although no significant differences were found in the types of infections between groups, patients with lower QFM values exhibited a higher prevalence of methicillin-resistant MRSE bloodstream infections. Potena et al. demonstrated that heart transplantation patients with lower QFM had a higher probability of developing clinical manifestations of infections; particularly, a cut-off of 100 IU/mL was found to discriminate the subgroup at higher risk of infection [30]. As demonstrated by Sood et al., even after liver transplantation, low QFM was associated with an increased susceptibility to infection and very low QFM (<30 IU/mL) was associated with death in patients who died while awaiting transplantation [31].

The QuantiFERON CMV assay (QCMV) is a new method that helps identify patients at increased risk of CMV replication. By measuring the release of interferon-γ (IFN-γ) from T lymphocytes in response to CMV, this test can predict the effectiveness of the body’s cellular immune response against CMV [32]. Some authors have discovered that a nonreactive QCMV is closely linked to an increased likelihood of CMV infection and/or disease. Additionally, the QCMV test allows us to pinpoint patients who are at risk of CMV replication and thus may require an extended period of prophylactic therapy [32]. As regards our cohort of patients, interestingly, there were no cases of CMV pneumonia in the study cohort. However, a statistically significant increase in CMV genome copies (<300 copies/mL) in BAL samples was noted in patients with lower QFM values and higher infection rates. This finding implies that lower QFM levels may reflect reduced control over CMV replication in the alveolar microenvironment rather than an increased risk of overt CMV disease. In a study by Douglas et al., the authors found no significant difference in IFN-γ levels between those patients with and without any detectable CMV viremia; IFN-γ levels were significantly lower in those with CMV viral load >1000 compared to those with a load <1000 or undetectable [28]. Very recently, Yoon et al. demonstrated that QFM at post-HSCT week 4 can be utilized to predict the risk and burden of early CMV infection in HSCT recipients, with a cutoff for predicting CMV infection of 86.95 IU/mL [33].

There was no statistically significant linear correlation between QFM values and the incidence of acute or chronic graft rejection, which was confirmed by literature data [28,29,30]. Nevertheless, patients with higher QFM levels showed a tendency toward acute rejection episodes requiring treatment, possibly due to heightened innate immune activation. This aligns with the observed higher neutrophilia in BAL samples at four months post-transplantation in patients with higher QFM values. While this neutrophilic response initially appeared to reflect increased innate immune activity, its significance diminished in subsequent follow-up, likely due to the immunomodulatory effects of azithromycin therapy.

The QFM values suggested by the assay manufacturer could not be appropriate to describe the immunoactivity of lung transplant patients, also, according to the study by Gardiner B. J. et al. [29]. In fact, most patients in this study had QFM values related to the median activity of the immune system (QFM between 15 and 1000 UI/mL), reducing the predictive value of the test. In previous studies of lung transplant patients, QFM was below 15 UI/mL only in the first months or in severe immunodepression. This evidence suggests that, in these patients, although on immunosuppressive therapy, the immune response was higher than normal people, probably precisely because of the immune stimulation graft. Using ROC curve analysis, we identified a threshold of <89.5 UI/mL as a marker of increased infection risk at 18 months post-transplantation but, due to the small population included in the study, it does not allow us to draw definitive conclusions and needs validation in a larger cohort.

Our study has several limitations. Firstly, the small population included in the analysis significantly limits the statistical power and generalizability of the findings; in addition, the retrospective nature of the study is another potential cause of potential bias. Nevertheless, given the small number of patients that annually undergo lung transplantation, our study is one of the few studies that evaluated QFM in lung-transplanted patients with a follow-up longer than one year after transplantation. Another limitation is only one determination of QFM; probably this single value is not enough to indicate changes in patients’ immune status during follow-up, especially in case of infections. The decision to include the QFM measurement at the 18th month after lung transplantation coincides with the last scheduled follow-up evaluation.

To overcome these limitations and to help physicians in the prognostic evaluation and therapeutic approaches to the patients, future approaches could integrate QFM results with clinical, laboratory, radiological, and genetic data in a deep-learning model as already used in other studies about different topics [34,35].

## 5. Conclusions

This study suggests that the QFM assay can help stratify lung transplant patients by infection risk and personalize follow-up care. However, its role in predicting rejection is less clear. Further multicenter studies with larger cohorts are needed to validate these findings and establish time-dependent cut-off values that consider the dynamic interaction between immune activation and immunosuppressive therapy.

Using time-specific QFM thresholds could enhance the management of infection-rejection balance in such patients. These thresholds may be adaptable for other solid organ transplants, leading to more personalized immunosuppressive regimens.

This study also suggests potential for therapeutic interventions targeting nonantigen-specific immunity. Modulating innate immune responses could address vulnerabilities, either by boosting immune activity to reduce infections or tempering hyperactivity to lower rejection risk.

In conclusion, the QFM test shows promise as a biomarker for monitoring immune status in lung transplant recipients, aiding in guiding immunosuppressive therapy and antimicrobial prophylaxis. However, its ability to predict graft rejection requires further investigation with larger patient cohorts to optimize its clinical application.

## Figures and Tables

**Table 1:** Patients’ characteristics and immunosuppressive therapy set in post-transplantation.

Population	HI	LI	*p* < 0.05
Number of patients	6	9	/
Age (years, ± SD)	48.67 ± 3.00	51.44 ± 4.48	0.654
Sex (Females: n, %)	5 (83.3%)	2 (22.2%)	0.04
Kind of transplant: Single Bilateral	0 (0%)6 (100%)	1 (11.1%)8 (88.9%)	0.435
Transplant for IPF	1 (16.7%)	2 (22.2%)	1.00
Transplant for NSIP	1 (16.7%)	1 (11.1%)	1.00
Transplant for CF	0 (0%)	2 (22.2%)	0.485
Transplant for COPD	0 (0%)	3 (33.3%)	0.228
Transplant for AAT deficiency	0 (0%)	0 (0%)	1.00
Transplant for PH	1 (16.7%)	0 (0%)	0.400
Transplantation for other causes	3 (50%)	1 (11.1%)	0.235
Average total number of infections	8.66 ± 0.88	2.44 ± 0.72	*p* < 0.001
Immunosuppressor 1			
Cyclosporin	4 (66.7%)	4 (44.4%)	0.608
Tacrolimus	2 (33.3%)	5 (55.6%)	0.608
Immunosuppressor 2			
Mycophenolate Mofetil	3 (50%)	7 (77.8%)	0.328
Mycophenolate Sodium	3 (50%)	2 (22.2%)	0.328

Abbreviation list: AAT, alpha 1 antitrypsin; CF, cystic fibrosis; COPD, chronic obstructive pulmonary disease; IPF, idiopathic pulmonary fibrosis; NSIP, nonspecific interstitial pneumonia; PH, pulmonary hypertension.

## Data Availability

The datasets presented in this article are not readily available due to technical limitations. Requests to access the datasets should be directed to the corresponding author.
